# Disordered eating behaviors and associated factors among secondary school adolescents in Harar town, eastern Ethiopia: cross-sectional study

**DOI:** 10.3389/fpsyt.2024.1459073

**Published:** 2024-12-18

**Authors:** Abainash Tekola, Lemma Demissie Regassa, Hiwot Berhanu, Miheret Mandefro, Samrawit Shawel, Obsan Kassa, Kedir Teji Roba

**Affiliations:** College of Health and Medical Science, Haramaya University, Harar, Ethiopia

**Keywords:** disordered eating attitude, eating disorder, unhealthy weight control behaviors, dieting, binge eating

## Abstract

**Background:**

Adolescent eating disorders impair physical and mental development and are associated with poor health outcomes in adulthood. However, there is little research on disordered eating in Ethiopia, particularly in the study area. As a result, the purpose of this study is to examine disordered eating behaviors and associated factors in secondary school adolescents in the study area.

**Methods:**

A school-based cross-sectional study was conducted among 1104 secondary school adolescents in Harar town from June 30 to July 15, 2022. Multistage cluster sampling was used, and data was collected using self-administered questionnaires. The eating attitude test-26 was used to assess disordered eating behaviors, and scores of 20 and above were considered to have disordered eating behaviors. A binary logistic regression analysis was done to identify factors associated with disordered eating behaviors and the statistical significance level was set at a p-value of 0.05.

**Result:**

In this study, 28.37% [95% CI (25.73%, 31.16%)] of the adolescents had disordered eating behaviors. Being female [AOR = 1.81, 95% CI (1.29, 2.53)], being from a mother, attending technical school [AOR = 3.61, 95% CI: (1.85, 7.04)], and having a higher-educated father [AOR = 1.55, 95% CI: (1.02, 2.36)] were significantly associated with disordered eating behaviors. Furthermore, desire to lose weight [AOR = 2.09, 95% CI (1.49, 2.93)], smoking [AOR = 3.64, 95% CI (2.24, 5.91)], emotional problems [AOR = 1.7, 95% CI (1.14, 2.55)], and body image dissatisfaction [AOR = 0.96, 95% CI (0.94, 0.97)] were associated with disordered eating.

**Conclusion:**

In Harar, almost three out of ten secondary school adolescents had disordered eating behaviors. Personal, behavioral, and socio-demographic factors were associated with disordered eating. Since the prevalence of disordered eating is significant at the study site, all relevant stakeholders should have to develop appropriate interventions that target modifiable factors to reduce the burden.

## Introduction

Eating disorders (ED) are psychological disorders characterized by abnormal or disturbed eating behaviors with or without compensatory behaviors, as diagnosed by the DSM-5 (1). Disordered eating behavior (DEB) is a condition characterized by the same characteristics but a lesser frequency or a lower level of severity as that of an eating disorder. Behaviors like food restriction, binge eating, excessive exercise, and the use of laxatives and diet pills are some examples of DEB (2).

Globally, 700 million people live with eating disorders, and global eating disorder prevalence increased from 3.4% to 7.8% between 2000 and 2018 (3). Various studies indicate an increase in the prevalence of disordered eating behaviors in the developing world (4, 5). It has been argued that “Western” communities with a higher prevalence of eating disorders tend to influence non-Western societies in a variety of ways, including an emphasis on thinness (6, 7). A study conducted among adolescents in various parts of the world shows different burdens of disordered eating. In Malaysia, 30% (8) 16.7% in Iran (9), 17.3% in Brazil (10), 28.8% in China (11), 15.20% in Turkey (12), 25.47% in Saudi Arabia (12), and 20% in Johannesburg (13). The previous study conducted in Addis Ababa, Ethiopia, from May to June 2015 revealed that the prevalence of disordered eating among high school adolescents was 8.6%. It shows a higher prevalence among female adolescents (5.9%) compared to males (2.6%). This study use eating attitude test 26 (EAT-26) with score of 20 or higher as cut cutoff point to assess disordered eating (14).

These complex disorders are thought to be caused by the interaction of behavioral, sociocultural, psychological, and environmental factors (15, 16). Female gender, age, self-perception of being overweight, intention to lose weight, and higher media message exposure were all found to be associated with disordered eating behaviors (9, 16–19).

Adolescents are the future generation of any country, and their nutritional needs are critical for the well-being of society. In Ethiopia, adolescents and youth aged 10 to 29 are estimated to constitute 42% of the total population (20). Eating disorders are one of the most significant problems in adolescents and are associated with a variety of metabolic and psychological symptoms. Of the psychological outcomes, it is associated with mental illnesses like depression, anxiety, impairments in the fulfillment of roles, and suicidality (21). Early adolescent disordered eating behaviors are also predictive of continued use of these behaviors as well as progression to a clinical eating disorder later in adolescence or young adulthood (22, 23).

Beside psychological problems, disordered eating increases the risk of physical comorbidities like diabetes, hypertension, back and neck pain, and chronic headaches (24). Disordered eating behavior is also one of the risk factors for the double burden of malnutrition, as dietary restraints such as dieting, appetite suppressants, and exercise for weight reduction increase the risk of obesity (24, 25). Although the exact mechanism underlying this is unknown, studies suggest that changes in metabolism, neuroendocrine adaptation, subjective appetite and change in gastrointestinal motility, cause obesity after engagement to those behaviors (26). Under nutrition can also occur in any individual engaging in disordered eating, regardless of weight status, especially during adolescence (an important period of physical and cognitive development that needs optimal nutrition) (27). Generally, eating disorders and milder disturbed eating behaviors are associated with both long-term and short-term consequences. From the short-term consequence, dizziness, nausea, weakness, and blurred vision, and in the long term, it leads to many negative medical outcomes, including poor metabolic control, increased frequency of diabetes-related complications, thyroid disorder, gastrointestinal disorder, fertility problems, and psychiatric comorbidity (28).

Even though disordered eating is well studied in developed countries, there is little evidence about disordered eating in Africa. In Ethiopia, there is a single study on those behaviors that was conducted seven years ago in Addis Ababa, but there is no study that shows the current prevalence of disordered eating. Beside this, there is no prevention program to address disordered eating in Ethiopia. As a result, it is important to know the current prevalence and potential risk factors to design appropriate prevention programs. Hence, this study may be used as an input for policymakers to develop effective prevention programs to address the problem. In addition to this, it may provide some insight on the current level of disordered eating to the community in the study area. Therefore, the purpose of this study is to investigate the prevalence of disordered eating behaviors and their associated factors among secondary school adolescents in Harar town in eastern Ethiopia.

## Materials and methods

### Study setting, design and participants

A school-based cross-sectional survey was conducted among secondary school adolescents in Harar, eastern Ethiopia, from June 30 to July 15, 2022. Harar is located 525 kilometers east of Addis Ababa (the capital of Ethiopia). There are six districts and 19 kebeles (the smallest administrative unit in Ethiopia). According to the 2007 Central Statistical Agency population census, the town’s total population is estimated to be 151,977 people. Harar had 15 secondary schools, seven of which were public and the remaining eight private. Harar had 11202 secondary school students in 2022, according to the Harar Town Educational Office report, with 6261 males and 4918 females.

Study design and population: This study employed a multicenter facility-based cross-sectional study design. The study population consists of all secondary school adolescents (aged 10 to 19) in Harar who were active and willing to participate during the data collection period. The study excluded adolescents enrolled in the night secondary school program.

### Sample size and sampling procedure

The sample size for this study was calculated using both single and double population proportion formulas. Sample size for specific objective 1 was calculated by using single population proportion formula by considering the following assumption.


$$\text{n}=\frac{{\text{z}}^{2}\text{pq}}{{\text{d}}^{2}}\text{ where z}=1.96$$

*p=expected prevalence of disordered eating among adolescents was 8.6% from previous study conducted in Addis Ababa* (14).


q=1−p, 1−0.086 = 0.914

d=4%, margin of error

$n={\left(1.96\right)}^{2}*0.916*0.086/0.04^{2}$ , = 207 and by considering design effect of 2 the sample size become 414. Adding 10% for non-response: 414 + 42 = 456 and the sample size for factors associated with disordered eating behaviors was calculated using Epi-Info software version 7.2.5.0 and taking into account the assumptions mentioned in **Table 1**. The sample size calculated using the double population proportion formula for being female was greater than the sample size calculated using the single population formula. As a result, the study’s minimum planned sample size was 817 people.

**Table 1:** Sample size determination for factors associated with DEB among secondary school adolescents.

Factors	% in exposed	% in non Exposed	AOR	Power	Sample size	Total with 5% NRR	Reference
Being female	12 (female)	6	1.75	80	778	817	(14)
Being overweight	43.2 (overweight)	20	3	80	142	149	(12)
Being vegetarian	61.9 (vegetarian)	23.2	4.8	80	60	63	(12)

To select a minimum of 817 adolescent students from Harar’s secondary schools, multistage cluster sampling were used. Harar towns had 15 secondary schools. Eight schools (four public and four private) were chosen by lottery. Following that, sections that had to be included were drawn at random from all sections in randomly selected secondary schools. Finally, all adolescents in randomly selected sections were included in the study. So while including all adolescents in selected section, the total number of adolescents in the total sections becomes 1104. Therefore the actual sample was 1104.

### Data collection methods

Structured, pretested, and self-administered questionnaires were used to collect data on socio-demographic factors, eating habits, environmental factors, personal factors, and disordered eating behaviors. Standardized self-report questionnaires were used to assess disordered eating, body image dissatisfaction, and emotional problems (29–31) and the rest of the questionnaires were developed by reviewing different literature. For data collection, four public health graduates and two supervisors were recruited. Data collectors administered a questionnaire to participants after obtaining consent. The participant then returned the questionnaire after answering each question. In addition, each participant’s anthropometric measurements were taken twice and recorded.

### Variables

There was one dependent variable in this study, which was disordered eating and the independent variables were, socio-demographic, personal, and environmental factors. Age, sex, age at menarche, and maternal educational status were socio-demographic factors. Peer pressure and high media message exposure were environmental factors, and body weight, body appearance satisfaction, perceptional body weight, desire to lose weight, emotional problems, and being vegetarian were personal factors addressed in this study.

#### Disordered eating behaviors

The Eating Attitude Test 26 (EAT-26), a highly reliable and valid test, was used to evaluate it. It has 26 items that have been divided into three factors by subscales. Dieting has 13 subscales in Factor I. Factor II includes six subscales for “bulimia and food obsession,” while Factor III includes seven subscales for “oral control.” Each response receives the following value for all items except the 26th: 3 points for “always,” 2 points for “often,” 1 point for “usually,” and 0 points for “sometimes, rarely, and never” items. Item number 26 received a different rating. 0 points for “always, often, and usually,” 1 point for “some time,” 2 points for “rarely,” and 3 points for “never.” As a result, a maximum score of 78 was assigned (29). This instrument was previously used in Ethiopia (14). The scale has very good internal consistency in this study (α =.88).

#### Body image dissatisfaction

The body part satisfaction scale was used. Face, upper torso, middle torso, lower torso, height, body weight, muscle tone, and overall appearance are among the eight items on the scale. On a 6-point scale, the scale was graded. One represents extreme dissatisfaction, two represents quite dissatisfaction, three represents some-what dissatisfaction, four represents some-what satisfaction, five represents quite satisfaction, and six represents extreme satisfaction. A higher score was thought to indicate greater satisfaction with the appearance. This scale was employed in Ethiopia (31). This scale has the highest internal consistency (α =.92) in this study.

#### Emotional problem

The Depression, Anxiety, and Stress Scale-21 (DASS-21), three self-reported scales designed to assess emotional states, were used to assess this. There are seven items on each of the three DASS-21 scales. The depression scale measures dysphoria, hopelessness, life devaluation, and self-deprecation, lack of interest, anhedonia involvement, and inertia. The anxiety scale measures autonomic arousal, skeletal muscle effects, situational anxiety, and subjective anxiety experience. The stress scale was sensitive to chronic, nonspecific arousal levels. It evaluates difficulty relaxing, nervous arousal, and being easily agitated, irritable, over reactive, and impatient. Scores for depression, anxiety, and stress were computed by adding the scores from the relevant items and multiplying the total by two (30). This test has previously been used in Ethiopia (32). The scale has the highest internal consistency (α =.91) in this study. The level of emotional problems is considered abnormal if the score is greater than 9, greater than 7, and greater than 14 for depression, anxiety, and stress, respectively (30).

#### Anthropometric measurement

Using an electronic portable scale, weight was measured to the nearest 0.1kg. Before each weight measurement, the scale was checked for a zero reading to ensure measurement accuracy. A portable studio meter was used to measure height in the standing position to the nearest 0.5 cm. The participant was instructed to stand without shoes, with their backs to the scale, heels together, and heads upright. The movable headboard was lowered until it made firm contact with the upper part of the participant’s head. The WHO growth reference from 2007 was used as a standard reference for categorizing adolescents based on BMI (body mass index) for age. BMI for age Z-score <−3 was classified as severely thin, ≥−3 and <−2 as thin, ≥−2 and ≤+1 as normal weight, >+1 and ≤+2 as overweight, and >+2 as obese (33).

### Operational definitions

#### Disordered eating

Was assessed using Eat-26 and a participant who have Eat–26 score of ≥ 20 was considered as having disordered eating (29).

#### Emotional problems

was assessed by DASS-21 scales. The pathological score was set above 9,7, and 14 For depression, anxiety, and stress domain respectively. Participant with at least one of those pathological score was considered as having emotional problem (30).

#### Exposure to media

A respondent who read newspapers, listened to the radio, watched television, or who have used at least one social media account in the last week before data collection was considered to be regularly exposed to that form of media, and those participants who have been exposed to at least one of those media was considered as having media exposure (34).

### Data quality control

To ensure the consistency of the data, the questionnaire was translated into Amharic and Afaan Oromo before being translated back into English. Data collectors and supervisors received one-day training. The questionnaire was pretested on 5% of the sample size (35) to ensure its suitability for the local context. To ensure reliability and reduce technical measurement errors, two different data collectors took weight and height measurements. Before each measurement, weight-measuring scales were standardized and calibrated to zero. The data collection process was monitored on a daily basis. Data were immediately checked for completeness at the data collection site. To reduce errors, data was entered by two different people. To control for confounding variables, multivariable analysis was used.

### Data processing and analysis

Before entering data, the data was cleaned, checked for completeness, and assigned a unique code. Data was entered into the EPI-DATA version 4.6.2 statistical software by two different individuals. It was then exported to STATA version 14 for further analysis. Anthropometric data were exported to WHO Anthro Plus software after calculating average height and weight to generate BMI for age Z-scores. The magnitude of disordered eating behaviors was calculated by adding the scores of each item on the EAT-26 and then categorizing the EAT-26 score as less than 20 or greater than 20. Participants with an EAT-26 score of 20 were considered to have disordered eating behavior. The categorical variables were described using descriptive statistics such as frequencies and percentages. Age, body part satisfaction score, and age of menarche were described using the median with an interquartile range (IQR). The magnitude of disordered eating behaviors across groups was calculated using two-way tables with measures of association.

To assess factors associated with disordered eating behaviors, bivariable and multivariable binary logistic regression analyses were used. The variance inflation factor (VIF) was used to test for multicollinearity among independent variables, and no significant multicollinearity was found (VIF <10). A box tid well test was performed to examine the linear relationship between body part satisfaction score and logit transformation of the dependent variable, and its value was greater than 0.5 (p value = 0.889). A box plot was used to check for the presence of extreme outliers, and there were none. After conducting bivariable analysis, those independent variables with a P value of less than or equal to 0.25 were entered into stepwise backward analysis. The Hosmer and Lemeshow test was used to determine the model’s goodness-of-fit, and it fit well (p = 0.3478). Adjusted odd ratio (AOR) and 95% confidence interval (CI) were estimated to determine factors associated with disordered eating behaviors, and statistical significance was declared at a p-value less than or equal to 0.05.

## Results

### Socio-demographic characteristics

The study included 1061 adolescents from 1104 in randomly selected sections, with a response rate of 96.1%. The reason for the non-response was absentees from the class despite repeated attempts to contact them. The median age of the participant was 17 with an IQR of 2 years. 572 (53.91%) of the total participants were females, and 867 (81.7%) were urban dwellers. In terms of family characteristics, 285 (26.86%) of adolescents’ mothers and 155 (14.61%) of fathers have no formal education. In this study, 724 (68.24%) of the participants came from public schools, and nearly one-third were in grade 9. In terms of family occupation, the majority of adolescents’ mothers (33.84%) were housewives, and the majority of their fathers (28.28%) were farmers (**Table 2**).

**Table 2:** Socio-demographic characteristics of the participants and their family, July 2022.

Variables	Categories	Frequency	Percentage
Sex	Male	489	46.09
	Female	572	53.91
Age	10-13	9	0.85
	14-16	672	63.34
	≥17	380	35.81
Residence	Urban	867	81.72
	Rural	194	18.28
Religion	Muslim	601	56.64
	Orthodox	364	34.31
	Protestant	82	7.73
	Others	14	1.32
Maternal educational status	No formal education	285	26.86
	Primary school	209	19.70
	Secondary school	290	27.33
	Technical/vocational	53	5.00
	Higher	224	21.11
Paternal educational status	No formal education	155	14.61
	Primary school	212	19.98
	Secondary school	303	28.56
	Technical/vocational	64	6.03
	Higher	327	30.82

### Nutritional status

There were 862 (81.24%) participants with normal body weight, 21 (1.98%) participants were severely thin, 107 (10.08%) were thin, 60 (5.66%) were overweight, and 11 (1.04%) were obese. In this study, 57 (11.66%) of males and 71 (12.241%) of females were underweight; on the other hand, 34 (6.95%) of boys and 37 (6.47%) of females were overweight.

### Personal characteristics

In this study, only 53.1% of the participants perceived that they had a normal body weight, and 762 (71.8%) were satisfied with their current body weight. In other words, nearly one-third (29.3%) of the participants wanted to lose weight. This study reveals that 252 (23.75%) of the participants have chewed chat. In this study, menarche occurred at the minimum age of seven, with a median age of 13 and an IQR of 2 years.

### Environmental factors

According to the findings of this study, one-third of adolescents (31.10%) felt pressured by their peers to choose a weight-management strategy. The majority of participants (95.95%) use at least one form of media, and more than two-thirds have used at least one of their social media accounts in the previous week (**Table 3**).

**Table 3:** Distribution of environmental factors among participants, July 2022.

Variables	Categories	Frequency	Percentage
Used at least one form of media	Yes	1,018	95.95
	No	43	4.05
Read magazines	Yes	587	55.33
	No	474	44.67
Watched television	Yes	927	87.37
	No	134	12.63
Listen radio	Yes	575	54.19
	No	486	45.81
Used social media	Yes	743	70.03
	No	318	29.97
Felt pressure from peer to decide on weight management	Yes	330	31.10
	No	731	68.90

### Magnitude of disordered eating behaviors

In this study, the prevalence of disordered eating behaviors was 28.37% [95% CI (25.73%, 31.16%)] and it varies with sex. It was 184 (32.20%) among females and 117 (23.93%) among males. In addition to this, disordered eating varies with school type, residence, maternal educational status, age of menarche, desire to lose weight, perceptional body weight, actual body weight, being vegetarian, feeling pressure from peers to control weight, smoking at least some days, and having emotional problems (**Table 4**).

**Table 4:** Distribution of DEB across participants, July 2022.

Variables	Categories	Frequency	Percentage
Sex	Female	184	32.20
	Male	117	23.93
School type	Public	238	32.87
	Private	63	18.69
Residence	Rural	70	36.08
	Urban	231	26.64
Maternal educational status	No formal	88	30.88
	Primary school	60	28.71
	Secondary school	79	27.24
	Technical/vocational	21	39.62
	Higher	42	18.75
Paternal educational level	No formal	46	29.68
	Primary school	59	27.83
	Secondary school	86	28.38
	Technical/vocational	26	40.63
	Higher	84	25.69
Perceptional body weight	Normal	129	22.91
	Under weight	107	30.06
	Overweight	53	43.80
	Obese	12	57.14
Body weight satisfaction	No	100	33.44
	Yes	201	26.38
Desire to lose weight	No	160	21.33
	Yes	141	45.34
Body weight	Normal	253	29.35
	Under weight	30	23.44
	Overweight	14	23.33
	Obese	4	36.36
Being vegetarian	Yes	47	43.52
	No	254	26.65
Emotional problem	No	43	15.30
	Yes	258	33.30
Peer pressure	Yes	130	39.39
	No	171	23.39
Age at menarche	>=11	280	27.86
	<11	21	37.50
Exposure to social media	Yes	294	28.54
	No	7	22.58

### Factors associated with disordered eating behaviors

To identify factors associated with disordered eating behaviors, bivariable and multivariable analysis were used. In multivariable analysis, all variables with a p value of 0.25 in bivariable analysis were included. A stepwise backward analysis was used to identify factors that were significantly associated with DEB. The odds of DEB were 81% [AOR = 1.81, 95% CI: (1.29, 2.53)] higher among females compared to males. Furthermore, the odds of DEB were more than three times higher [AOR = 3.61, 95% CI: (1.85, 7.04)] among adolescents from mothers with a vocational educational status compared to adolescents from mothers with no formal education. It was 55% [AOR = 1.55, 95% CI (1.02, 2.36)] higher among adolescents whose fathers have a higher level of education compared to others. With a one-unit increase in body part satisfaction score, the odds of disordered eating behavior were reduced by 4% [AOR = 0.96, 95% CI (0.94, 0.97)]. When compared to other adolescents, the odds of disordered eating behaviors were two times higher [AOR = 2.09, 95% CI (1.49, 2.93)] among adolescents who had a desire to lose weight. This study also discovered a link between disordered eating and emotional problems [AOR = 1.7(1.14, 2.55)]. When compared to those who did not smoke, the odds of DEB were more than three times higher [AOR = 3.64, 95%CI (2.24, 5.91)] among adolescents who smoke at least some days (**Table 5**).

**Table 5:** Factors associated with DEB among secondary school adolescents in Harar town, eastern Ethiopia. .

Variables	DEB (%)		Crude odd ratio (95% CI)	AOR (95% CI)
	Yes	No		
Sex				
Male	117 (23.93)	372 (76.07)	1.00	1.00
Female	184 (32.20)	388 (67.83)	1.51 (1.15,1.98)^**^	1.81 (1.29, 2.53)^**^
Maternal educational status				
No formal	88 (30.88)	197 (69.12)	1.00	1.00
Primary school	60 (28.71)	149 (71.29)	.90 (.61,1.33)	0.96 (0.60, 1.55)
Secondary school	79 (27.24)	211 (72.76)	.84 (.58, 1.20)	1.19 (0.81, 1.74)
Technical/vocational	21 (39.62)	32 (60.38)	3.41 (1.86,6.24)^**^	3.61 (1.85, 7.04)^**^
Higher	42 (18.75)	182 (81.25)	.516 (.34,.78)	0.92 (0.52, 1.65)
Paternal educational level				
No formal	46 (29.68)	109 (70.32)	1.00	1.00
Primary school	59 (27.83)	153 (72.17)	.91 (.58, 1.44)	1.10 (0.65, 1.87)
Secondary school	86 (28.38)	217 (71.62)	.94 (.61, 1.44)	1.36 (0.89, 2.08)
Technical	26 (40.63)	38 (59.38)	1.6 (.88, 2.97)	1.59 (0.82 3.10)
Higher	84 (25.69)	243 (74.31)	.82 (.54, 1.25)	1.55 (1.02 2.36)^*^
Emotional problem				
No	43 (15.30)	243 (84.97)	1.00	1.00
Yes	258 (33.30)	517 (66.70)	2.8 (1.97, 4.03)^**^	1.70 (1.14, 2.55)^**^
Desire to lose weight				
No	160 (21.33)	590 (78.67)	1.00	1.00
Yes	141 (45.34)	170 (54.66)	3.06 (2.3, 4.1)^**^	2.09 (1.49, 2.93)^**^
Smoking				
No	230 (24.26)	71 8 (75.74)	1.0	1.0
Yes	71 (62.83)	42 (37.17)	5.27 (3.50,7.95)^**^	3.64 (2.24, 5.91)^**^
Body part satisfaction score---- ---			0.94 (0.93,0.96)^**^	0.96 (0.94, 0.97)^**^

* (p value 0.05-0.01); ** (P value < 0.01).

## Discussion

In this study, the prevalence of disordered eating behaviors was 28.37%. Being female, family educational status, body image dissatisfaction, desire to lose weight, having emotional problems, and smoking at least some of the time were associated with disordered eating behaviors. Currently, Ethiopia is working on adolescents by considering them as the second window of opportunity to break the intergenerational cycle of malnutrition, and disordered eating is found to be one of the causes of both under nutrition and over nutrition (36). Hence, to improve adolescent nutrition, emphasis has to be given to this health problem. Therefore, health promotion activity has to be done to reduce the burden.

The prevalence of DEB in this study was higher than previous studies conducted in Africa, in which the prevalence of disordered eating ranged from 7.1% to 20% (13, 37). It was also much higher than the previous study conducted in Ethiopia from May to June 2015 among 836 high school adolescents in Addis Ababa, which revealed that the prevalence of disordered eating was 8.6%. This inconsistency in prevalence could be due to differences in study periods, as those studies were conducted a minimum of five years ago, and there is no recent study that shows the current prevalence of disordered eating in African countries, including Ethiopia. With this variation in study period, there is an increase in the level of social media utilization, which might expose them to the western idea of being thin as a sign of attractiveness, which may increase the risk of disordered eating (38). In addition to this, it may expose adolescents to many pictures, messages and influencer that encourage unachievable body and beauty ideals (39). Those messages from social media may stress how important is to have a socially acceptable weight status at the period they are undergoing to find their identity (40). Beside this, the rise in prevalence could be due to the rise in overweight and obesity (35). As some interventions that aim to reduce overweight and obesity may impose on adolescents the practice of engaging in these practices. For example, strategies to prevent obesity (monitoring intake and portion control) might unintentionally promote shape concerns and disordered eating (41). Since the prevalence of disordered eating at the study site is significant, health professionals have to screen for these behaviors to reduce the burden before it possibly progresses to eating disorders. Along with this, schools have to consider this important health issue during their school-based social and behavioral change communication.

Despite differences in sample size, study period, and assessment tools, it was comparable to studies conducted in Malaysia (30.1%) (8), China (28.8%) (11), and Jeddah (32.9%) (42). It does, however, show a lower prevalence compared to a study conducted in the United Arab Emirates (43) and Australia (19). This disparity could be attributed to cultural differences. In Ethiopia, being fat was taken as a sign of being in a high economic class; therefore, most of the population discourages being slim, contrary to developed countries that take thinness as a sign of attractiveness. It can also be due to differences in assessment tools. The study conducted in Australia used an eating disorder examination questionnaire, and we used Eat-26 to measure disordered eating (19).

Being female was found to be significantly associated with disordered eating behaviors in this study. This is consistent with the findings of the study conducted in Israel (44) and a previous study conducted in Addis Ababa, Ethiopia (14). This could be because girls tend to gain body fat during adolescence, which causes a change in physical appearance and causes them to deviate from western societal ideas that define slim women as attractive. As females react more strongly to changes in body appearance than males, they may engage in poor eating habits and poor weight management (23, 45). There is also cultural influence that encourage females to engage in such behaviors. In Ethiopia it is more expected from females to be more beautiful in comparison to males and this may force female to engage in such behavior to reach the currently accepted sign of beauty that is being slim. It is also well documented that there is reduction in self-stem among female and rise in self-stem among males during adolescence. This lower self-stem with weight gain during adolescents may enforce them to engage in such behavior (40). Therefore, more attention has to be paid to female adolescents who are at risk of disordered eating.

In this study, there was an association between disordered eating and parental educational status. According to this study, the odds of DEB were higher among adolescents whose mothers had a vocational educational status and adolescents whose fathers had a higher educational level compared to those whose parents had no formal education. This finding contradicted a previous study conducted in Ethiopia, which discovered that adolescents with primary and secondary school education were less likely to develop disordered eating attitudes than adolescents with no schooling (14). However, this study is consistent with the studies conducted in China (46) and Sweden (47). This could be due to the fact that parents with a high educational level may nurture and control the eating habits of their children more than parents with no formal education. This nurturing as well as over-controlling eating habits of adolescents may expose adolescents to being engaged in those behaviors (48).

DEB was found to be significantly associated with the desire to lose weight in this study. This is consistent with the findings of a study conducted in Israel (44). This could be because adolescents who want to lose weight may be more concerned about their weight than others, and this high concern about body weight may expose them to engaging in those behaviors (49). Hence, emphasis has to be given to those adolescents who want to lose weight. Furthermore, health professionals have to consider this issue while counseling adolescents on weight reduction.

DEB was also more common in adolescents who had emotional problems. This is consistent with the findings of a survey conducted in Malaysia, which found that disordered eating among adolescents was associated with emotional problems (8). This can be due to the fact that adolescents with disordered eating may feel guilt, which may create emotional problems for them. Another explanation can be derived from the escape theory, in which people tend to escape negative emotion through overeating and believe that eating can relieve negative emotion (50) or it could be due to concomitant occurrence of psychiatric problems (51, 52) Therefore, health professionals have to screen adolescents with emotional problems for this behavior.

Body image dissatisfaction was found to be significantly related to DEB in this study. This is consistent with the findings of a study conducted in China and Brazil (11, 53). This may be due to the current rise in acceptance of being thin among females and being muscular as the ideal body image among males. This idea may pressure them to have this ideal body shape, even though they have a normal appearance. To reach this ideal body shape, they may engage in these behaviors (23). Therefore, to reduce the burden, adolescents have to be counseled on the scientifically acceptable body weight as well as ways to manage body weight in a way that doesn’t threaten their health.

This study also found that adolescents who smoked at least once a week had a higher risk of DEB. This is consistent with the findings of a study conducted in the United States of America and the State of Mexico (54, 55). This can be explained as those adolescents with disordered eating may engage in substance use to avoid awareness of unpleasant emotional experiences that happen due to disordered eating (56). Additionally, some studies found that adolescents who have already engaged in unhealthy behaviors are at increased risk for smoking; this can be due to the use of smoking as a means of appetite suppressant (57). Therefore, clinicians have to screen those adolescents engaged in smoking for disordered eating because they may be using smoking to suppress their appetite. In addition to this schools have to give priority to both disordered eating and smoking during the school based social and behavioral change communication that is currently implemented nationally to improve adolescent nutrition.

This study has a remarkable strength as it shows the current prevalence of this neglected but important public health problem in Ethiopia. This study shows the current prevalence of disordered eating behaviors among adolescents (the most vulnerable group for disordered eating behaviors) in the study area. Beside this, the study includes both female and male adolescents, which provides valuable awareness about DEB in both genders. It has also identified factors that determine disordered eating; therefore, health professionals may act on those factors to reduce the burden.

Despite its strength, this study has limitations. Some of them are since it is a cross-sectional study, it does not show the temporal relationship between the independent and dependent variables. In addition to this, since we have collected our data through a self-administered questionnaire, some participants may not understand our questions. Beside this, most of the assessed variables, such as disordered eating, body image dissatisfaction, eating habits, emotional problems, smoking, chewing chat, alcohol consumption, FCS, and socioeconomic status, may also be subject to social desirability bias. There was also a possibility of recall bias since the questionnaire for most of our variables, like DASS21, food consumption score, and exposure to social media, was based on recall. In addition to this, it is difficult to generalize the results to the community, as adolescents who do not attend school were not included in this study. Beside this, the study shows only the non-clinical aspect, as it used the EAT-26 scale, which doesn’t diagnose eating disorders. Therefore, future research on eating disorders as well as the consequence disordered eating is recommended. Despite this limitation, this study can be used as an input with other similar studies in conducting systematic reviews and meta-analysis to produce a pooled estimate, especially for low-income countries, as there is little evidence about the problem in these countries, including Ethiopia. It can also be used as base-line information for further nutritional study.

## Conclusion

Approximately three out of ten secondary school adolescents in Harar town had disordered eating behaviors, which were significantly related to personal, behavioral, and socio-demographic factors. Sociodemographic factors such as gender and family educational status, as well as other factors such as a desire to lose weight, body image dissatisfaction, emotional problems, and smoking, were found to be significantly associated with disordered eating in this study. Since the prevalence of disordered eating is significant at the study site, all relevant stakeholders, have to develop appropriate interventions that target all modifiable factors in order to reduce the burden. We also recommend parents to monitor their children’s eating habits in order to detect and treat disordered eating early before it possibly progresses to an eating disorder.

## Data Availability

The original contributions presented in the study are included in the article/supplementary material. Further inquiries can be directed to the corresponding author.
